# Saturated Alcoholic Tincture of Eupatorium Perfoliatum

**Published:** 1840-07

**Authors:** 


### SATURATED ALCOHOLIC TINCTURE ON EUPATORIUM PERFOLIATUM.

When in Galliopolis, Ohio, we were informed by Dr. Hibbard, that he is in the habit of treating ague and fever with this tincture. He gives it in teaspoonful doses several times during the intermission. It is intensely bitter, and neither purges, vomits nor sweats the patient. He has not found the decoction to answer the same purpose. He administers a decoction of Aristolochia Sepentaria at the same time. The Doctor assured us, that he had found this treatment successful in a great number of cases; and it might, he thinks, be made to supercede in a great degree the use of the sulphate of Quinine.

